# A data-driven approach to clinical decision support in tinnitus retraining therapy

**DOI:** 10.3389/fninf.2022.934433

**Published:** 2022-09-28

**Authors:** Katarzyna A. Tarnowska, Zbigniew W. Ras, Pawel J. Jastreboff

**Affiliations:** ^1^School of Computing, University of North Florida, Jacksonville, FL, United States; ^2^Computer Science Department, University of North Carolina, Charlotte, NC, United States; ^3^Polish-Japanese Academy of Information Technology, Warsaw, Poland; ^4^Department of Otolaryngology—Head & Neck Surgery, School of Medicine Emory University, Atlanta, GA, United States

**Keywords:** clinical decision support systems, tinnitus, knowledge-based systems, knowledge discovery, action rules, tinnitus retraining therapy

## Abstract

**Background:**

Tinnitus, known as “ringing in the ears”, is a widespread and frequently disabling hearing disorder. No pharmacological treatment exists, but clinical management techniques, such as tinnitus retraining therapy (TRT), prove effective in helping patients. Although effective, TRT is not widely offered, due to scarcity of expertise and complexity because of a high level of personalization. Within this study, a data-driven clinical decision support tool is proposed to guide clinicians in the delivery of TRT.

**Methods:**

This research proposes the formulation of data analytics models, based on supervised machine learning (ML) techniques, such as classification models and decision rules for diagnosis, and action rules for treatment to support the delivery of TRT. A knowledge-based framework for clinical decision support system (CDSS) is proposed as a UI-based Java application with embedded WEKA predictive models and Java Expert System Shell (JESS) rule engine with a pattern-matching algorithm for inference (Rete). The knowledge base is evaluated by the accuracy, coverage, and explainability of diagnostics predictions and treatment recommendations.

**Results:**

The ML methods were applied to a clinical dataset of tinnitus patients from the Tinnitus and Hyperacusis Center at Emory University School of Medicine, which describes 555 patients and 3,000 visits. The validated ML classification models for diagnosis and rules: association and actionable treatment patterns were embedded into the knowledge base of CDSS. The CDSS prototype was tested for accuracy and explainability of the decision support, with preliminary testing resulting in an average of 80% accuracy, satisfactory coverage, and explainability.

**Conclusions:**

The outcome is a validated prototype CDS system that is expected to facilitate the TRT practice.

## 1. Introduction

### 1.1. Tinnitus

Tinnitus is a highly prevalent and frequently severely impairing hearing disorder with a worldwide impact. Often described as “ringing in the ears”, tinnitus is the sensation of sound perception without an external sound source—“phantom auditory perception” (Jastreboff, 1990). The U.S. Centers for Disease Control estimates that nearly 15% of the general public—over 50 million Americans—experience a form of tinnitus. In addition, close to 90% have experienced at least temporary tinnitus, making it one of the most common health conditions in the United States. While about 20 million people struggle with burdensome chronic tinnitus, 2 million have extreme and debilitating cases (American Tinnitus Association, 2018). There are millions of general practice consultations every year where the primary complaint is tinnitus, equating to a major burden on healthcare services. Tinnitus has been the #1 claimed service-related disability for the American Veteran Administration for more than a decade (US Department of Veterans Affairs, 2019). Chronic disabling tinnitus has a devastating impact on the quality of life and psychosocial aspects of those affected (Makar et al., 2017). The disorder has a considerable heterogeneity and no single mechanism is likely to explain the presence of tinnitus in all those affected. Tinnitus can be associated with head and neck injuries, hearing loss, acoustic neuromas, drug toxicity, ear disease, and depression (Savage and Waddell, 2014).

### 1.2. Treatment of tinnitus

The heterogeneity and current limited knowledge about the pathophysiology of the different forms of tinnitus are reasons that hamper the identification of good candidates for an effective pharmacological treatment for tinnitus. Despite its growing prevalence and often-devastating effects, tinnitus remains a severely underfunded condition. There are no Food and Drug Administration (FDA) approved drugs available, and the quest for a new treatment option for tinnitus focuses on important challenges in tinnitus management (Swain et al., 2016). Clinical management strategies include counseling (education and advice), sound enrichment using ear-level sound generators or hearing aids, tinnitus masking, relaxation therapy, cognitive behavior therapy (CBT), and tinnitus retraining therapy (TRT) (Makar et al., 2017). Although a variety of therapeutic interventions are available, the complexity of tinnitus makes the management of the condition challenging. Evaluating results in the field of tinnitus is a difficult task, as no objective tinnitus measurement exists. It means there is no objective method for detecting the presence and the extent of tinnitus.

### 1.3. Tinnitus retraining therapy

During the last decades, advances in neuroimaging methods and the development of an animal model of tinnitus have contributed to an increasing understanding of the neuronal correlates of tinnitus (Langguth, 2015). TRT is a clinical implementation of the neurophysiological model of tinnitus (Jastreboff and Hazell, 2004). It is the habituation therapy used for the management of chronic subjective tinnitus. It includes counseling (TC) during structured sessions in combination with sound therapy (ST) to reduce the patient's tinnitus-evoked negative reaction to, and awareness of, tinnitus. ST sound stimulation is performed with low-level broadband sound generators and aims to mask tinnitus at the sound perception level. By reducing the tinnitus perception, TRT successfully helps patients to achieve control over their tinnitus, live a normal life, and participate in everyday activities (Reddy et al., 2019). Clinical studies confirm that TRT is an effective and robust treatment for chronic decompensated tinnitus (Zhao and Jiang, 2018; Nemade and Shinde, 2019). The majority of published clinical studies indicate TRT offers notable help for about 80% of patients and the severity of tinnitus decreases in a clinically significant and persistent manner. Furthermore, TRT offers an approach to treat other hearing disorders: hyperacusis, which is reduced tolerance to sounds, phonophobia, which is the fear of sound, and misophonia, increased sound sensitivity (Jastreboff and Jastreboff, 2000). TRT, although effective, is a complex treatment and must be highly individualized. Counseling and teaching are tailored to the needs of the patient, and therefore, they cannot be performed as group therapy (Jastreboff and Jastreboff, 2006). Sound therapy involves different types/models of instruments, and they must be fitted optimally at the “mixing point” to achieve habituation in the most effective manner (Jastreboff and Jastreboff, 2006). Because TRT has to focus on the individual needs and profile of a patient, it consequently requires significant time involvement of the personnel. Although promising, it is expensive and spans from several months to a couple of years. Despite its high effectiveness and international recognition, the therapy is not widely offered, mainly due to a lack of expertise and experience in its delivery. The main obstacle to the widespread adoption of this technique is a lack of trained and experienced audiologists.

### 1.4. Tinnitus data analytics

Data-driven approaches have the potential to reveal novel insights into tinnitus heterogeneity. However, there are limitations in data-driven studies for tinnitus management proposed so far. Most efforts involve applying traditional statistical methods, such as correlation and regression (Langguth et al., 2017). New forms of discovery *via* machine learning and big data methods have not been widely investigated. Data mining/machine learning methods proposed on tinnitus data were mostly confined to association analysis, predictive modeling, and clustering analysis. However, these studies were limited in terms of analyzed variables or provided inconclusive results (Anwar, 2013; van den Berge et al., 2017). The status quo of tinnitus data analytics lacks the application of discovery methods for actionable and personalized knowledge needed by medical practitioners. The outcomes are not analyzed with regard to treatment methods in order to seek actions leading to improvement. Also, the temporality of data is not considered. So far, data analytics efforts focus on variables describing psychoacoustic measures of tinnitus. These measures, although routinely obtained in many clinics and as part of research studies, have not been validated for being diagnostic, prognostic, discriminative, or responsive (Henry, 2016; Watts et al., 2018). Medical history and evaluation, review of the patient's medications, and assessment of an individual's distress or handicaps are also crucial for effective diagnosis and treatment (Kari et al., 2010). Finally, most research efforts conclude by presenting analytics without any further developments in the decision support tool. No integration into health IT systems nor plans on how to utilize the findings in clinical decision-making is currently being proposed. To date, this research is the first to propose a decision support system for TRT.

### 1.5. Technological perspectives on tinnitus

The postal survey of general practitioners (GPs) concluded that there was a substantial discrepancy between the scientific and technological perspectives on the management of tinnitus and the actual day-to-day practice in the primary care setting (Hall et al., 2011). Many GPs expressed an unmet need for a specific and concise training on tinnitus management. Low satisfaction with available treatment options was unequivocally mentioned by both GPs and ENTs (ear-nose-throat specialists) from all developed countries investigated by Hall et al. (2011). The results of that survey highlight the need for an effective therapy option, particularly for chronic subjective tinnitus. Despite a variety of options, the low success of the available tinnitus treatment options leads to the frustration of physicians and patients alike. Effective therapeutic options with guidelines about key diagnostic criteria are urgently needed.

## 2. Materials and methods

**Figure 1:**
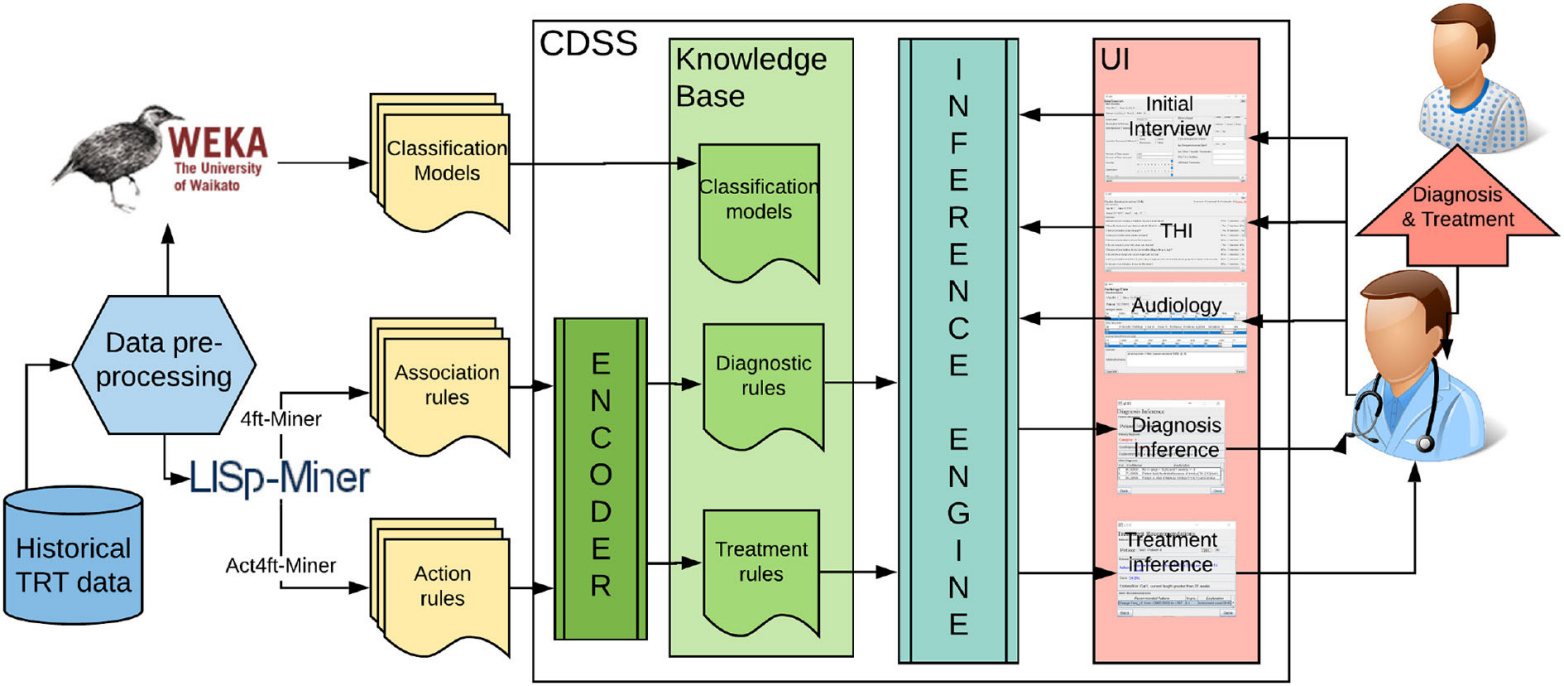
The architecture of the proposed data-driven clinical decision support system for tinnitus diagnosis and treatment.

Clinical decision support (CDS) is a process for enhancing health-related decisions and actions with pertinent, organized, clinical knowledge, and patient information to improve health and healthcare delivery. Systems, known as clinical decision support systems (CDSS), offer intelligent support for human-oriented diagnosis and treatment of patients. “CDS provides clinicians, staff, patients, or other individuals with knowledge and person-specific information, intelligently filtered or presented at appropriate times, to enhance health and healthcare” (Osheroff et al., 2007). They were proposed for various diseases, including traumatic brain injury, diabetes, Parkinson's disease, and other health-related decisions such as drug dosing (Ciecierski, 2013; Nielsen et al., 2014; Fartoumi et al., 2016; Torrent-Fontbona and López, 2019). Yet, nobody developed a clinical decision support system for tinnitus management. It was hypothesized that DSS can improve the accuracy and time efficiency of tinnitus management, but a design or implementation of such a system was not attempted (Thompson et al., 2007; Anwar, 2013). Within this research, we proposed a knowledge-based clinical decision support system (refer to Figure 1). The knowledge base is developed with validated models extracted from data mining experiments.

### 2.1. Knowledge discovery methods

The proposed knowledge discovery from tinnitus data, as opposed to previous research in this area, provides multidimensional evaluation beyond the psychoacoustic characteristics of tinnitus. Since the clinical data available describes temporal changes in tinnitus score and particular areas of a patient's life affected, it is possible to perform an analysis of changes and increased involvement in life activities that were previously prevented or interfered with by tinnitus (or hyperacusis). This approach will help in a better understanding of complex auditory, psychological, and medical conditions and aid in selecting the most significant variables to consider in TRT. We propose a variety of data mining methods to extract novel knowledge about TRT diagnosis and treatment. This includes predictive and descriptive models, which are extracted from the pre-processed and transformed data. The following software was used for knowledge discovery:

- WEKA—Open-source data mining software which offers a wide choice of algorithms for feature selection and for prediction as well as a user-friendly interface and feature to build a complete “knowledge flow” (Bouckaert et al., 2014). It also allows using Java API to embed machine learning models into a Java program.
- LISp-Miner—An academic system that offers exploratory data analysis, including modules for association rule discovery (4ft-Miner) and action rule discovery (Ac4ft-Miner) (Simunek, 2014).

#### 2.1.1. Dataset

**Figure 2:**
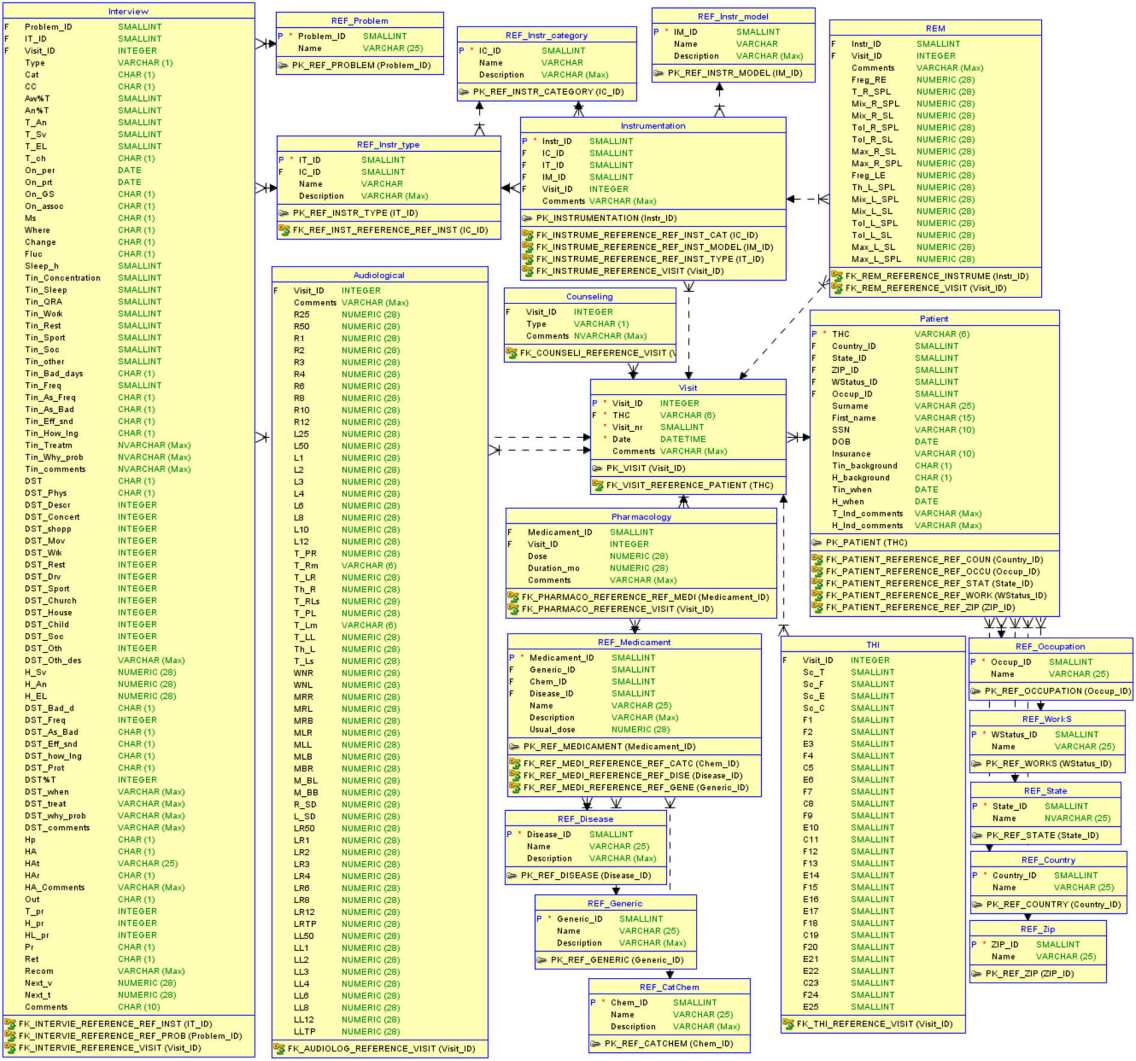
The relational database structure to store tinnitus-related data.

To evaluate our data-driven approach to building CDSS, we use clinical data collected at the Tinnitus and Hyperacusis Center of Emory University School of Medicine. The dataset contains records of tinnitus patients and the records for their sequential visits to the clinic. The dataset was collected over a period of several years and describes 555 unique patients and 3,000 visits in total. The raw data resides in 11 separate tables describing demographics, interview response, audiological measurements, pharmacology, additional medical evaluation, and visits. The visit data contains treatment methods applied by the physician at the visit (sound therapy with instruments/counseling, real ear measurements to help fit the instruments) along with the measure of the treatment progress using the Tinnitus Handicap Inventory (THI). The raw data were exported to the relational database system to ensure the structure, consistency, and integrity of the data (refer to Figure 2).

#### 2.1.2. Data pre-processing and feature selection

Various data-preprocessing techniques were applied to cleanse the data and handle real-life data issues, such as inconsistencies, incompleteness, duplication, and other problems. Data cleansing removed all inconsistencies, such as missing values, outliers, and duplicate data (e.g., duplicate visit numbers for the same patient). To handle missing data in the total score of the tinnitus handicap inventory (THI), an algorithm for data imputation was developed and validated. Additional transformations were applied, such as alphanumeric to numeric encoding, aggregation, and handling data temporality. Feature selection was proposed to reduce the data to a manageable and relevant size. Only the most relevant variables were involved in developing an analytical model. A more detailed description of the challenges with the real-world data and applied data-preprocessing methods to mitigate those can be found in our previous publication (Tarnowska et al., 2017).

#### 2.1.3. Feature extraction

Additional features describing the patient and characteristics of tinnitus were developed from the text attributes to make the dataset more suitable for machine learning:

- Tinnitus background: *STI* (stress-induced), *NTI* (noise-induced), *HLTI* (hearing-loss-induced), *DETI* (depression-related), *AATI* (auto accident-related), *OTI* (surgery-related), and *OMTI* (induced as a symptom of another medical condition).
- Temporal features: *DTI* (date tinnitus induced), *AgeInd* (the patient's age when tinnitus induced), *AgeBeg* (the patient's age when treatment began), binary features denoting how many days/weeks/months/years ago the hearing problem started.
- Binary attributes that represent the intake of medication.
- Attributes that keep track of a patient's improvement over time: *ChTsc* (change in the THI's total score from the previous visit) and *PerChTsc* (relative change measuring the percentage change in the THI's total score from the previous visit).

A comprehensive list of the attributes from the clinical database, as well as extracted features, can be found in our previous publication by Tarnowska et al. (2017).

#### 2.1.4. Predictive models

The first type of machine learning applied is supervised machine learning to build predictive models. The goal is to build an analytical model predicting a target measure of interest. In our domain, it is the category of the hearing problem, which determines the TRT treatment protocol. TRT protocol differentiates the following five categories, which differ in further treatment protocol: C0 (tinnitus minimal problem), C1 (tinnitus significant problem), C2 (tinnitus significant and hearing loss present), C3 (tinnitus irrelevant, hyperacusis significant), and C4 (prolonged tinnitus/prolonged exacerbation of hyperacusis). The proposed classification model, built using supervised machine learning methods, is used to predict the category of an unseen patient under consideration. The following ML algorithms in WEKA are used for classification models: tree-based J48, random forest, and probabilistic-based Naive Bayes.

#### 2.1.5. Descriptive models

The goal is to extract valid and useful medical patterns in tinnitus diagnosis and treatment. The patterns describe patients' diagnosis/treatment and are used to develop the domain knowledge for TRT. The descriptive methods used in this research include association rules and action rules. Rules are characterized by statistical measures quantifying their strength. Support and confidence are two key measures to quantify the strength and relevance of a rule. The support reflects the usefulness of a rule and confidence—its certainty. To find the significant associations, support and confidence must be set at a certain minimum threshold value (usually 1% for support, and 80% for confidence).

##### 2.1.5.1. Association rules for diagnosis in TRT

The TRT diagnosis is to be supported by the descriptive models based on the association (decision) rule discovery, as supplemental to predictive models.

**Figure 3:**
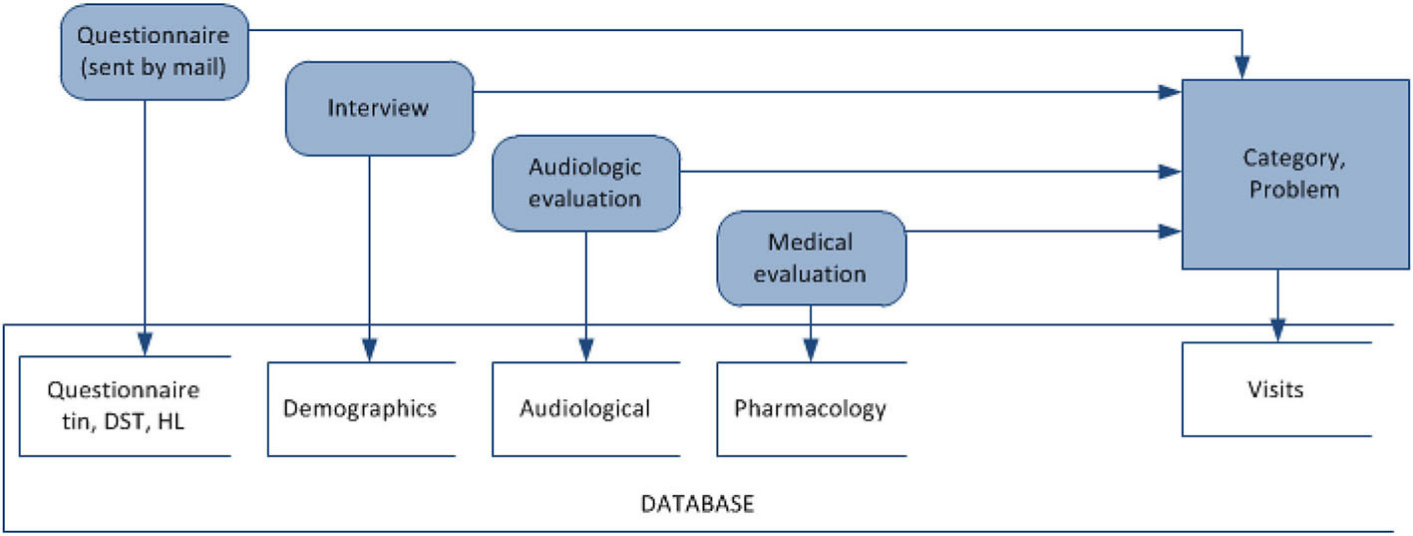
Mining associations between questionnaire and interview answers, audiology variables, medications, and category of a hearing problem for decision support in diagnosis in TRT.

A **decision rule** is a rule *r* in the form (ϕ ⇒ δ), where ϕ is called *antecedent* (or assumption), and δ is called *descendant* (or thesis). Each rule is characterized by *support* and *confidence*. *Support(r)* is defined as the number of objects matching the rule's antecedent. *Confidence(r)* is the relative number of objects matching both the rule's antecedent and descendant of the rule. The data mining experiments for decision rule discovery were modeled after the TRT diagnosis process, which involves an initial interview, audiology and medical evaluation (refer to Figure 3). Association rules mining aims at detecting frequently occurring associations between variables in TRT. Accordingly, associations between audiological measurements, demographics, questionnaire responses, pharmacology, and the category of tinnitus were extracted using LISP-Miner software for data mining (Simunek, 2014).

##### 2.1.5.2. Action rules for treatment in TRT

The concept of an action rule was first proposed by Ras and Wieczorkowska in 2000 (Ras and Wieczorkowska, 2000), and since then, its application was proposed, among others, for business, medicine, and music indexing (Ras and Wieczorkowska, 2000, 2010; Wasyluk et al., 2008; Tarnowska et al., 2020). Action rules are especially promising in the field of medical data, as a doctor can examine the effect of treatment decisions on a patient's improved state. This technique is also particularly useful for building knowledge-based decision support systems.

**Figure 4:**
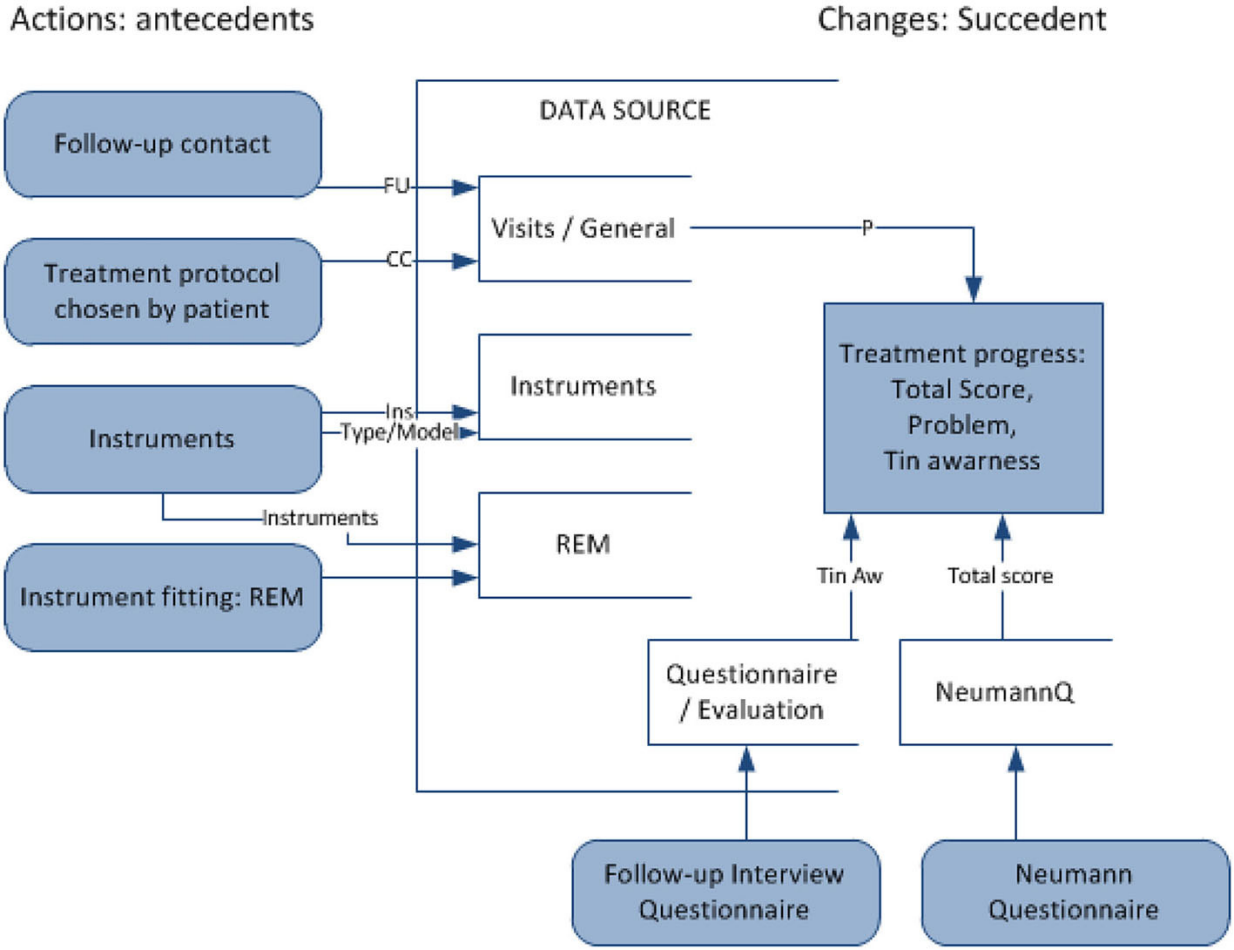
Mining action rules for changes in the type of counseling and tuning sound generators for decision support in TRT treatment.

**Action rule** *r* is a term [(ω)∧(α → β) ⇒ (θ → ψ)], where (ω∧α) ⇒ θ and (ω∧β) ⇒ ψ are classification rules, ω is a conjunction of stable attribute values, (α → β) shows changes in flexible attribute values, and (θ → ψ) shows the desired effect of the action. In this domain, it is proposed to apply action rules to recommend effective methods of treatment in TRT (refer to Figure 4). Such rules, extracted from large sets of data, represent actions to undertake (e.g., treatment methods) to improve the defined state (e.g., tinnitus awareness) when specified conditions hold (e.g., the current patient's state and profile). Action is understood as changing certain (“flexible”) variables to achieve the desired results. The purpose is to analyze data to seek specific actions to enhance the decision-making process. Action rules applied for TRT will suggest, with a certain confidence, the most effective treatment method for an individualized profile of a patient (defined by “stable” attributes), at a particular time (considering temporal variables).

### 2.2. Knowledge base

Within this research, we propose a knowledge-based clinical decision support system. The knowledge is developed with models extracted from knowledge discovery experiments. These experiments yield a vast number of diagnostic and treatment patterns. These are general clinical rules already known by experts, or they represent novel and unknown patterns useful for future diagnosis/treatment. We propose interpreting and validating the results from analytical modeling with clinical expertise and including only validated patterns with the highest confidence in the framework of the built system.

#### 2.2.1. Knowledge translator (encoder)

The goal of the Knowledge Translator (“Encoder” in Figure 1) component is to automatically encode the rule-based knowledge from the output files of data mining software into the CDSS knowledge base. The knowledge translating procedure reads rules one by one, parses, interprets them, adds the explanation in natural language, and encodes them into the syntax used in the knowledge base of CDSS. The rules encoded in KB are “if-then” like statements and each rule encodes a small piece of the expert's knowledge available through the dataset. The pseudocode for the knowledge translating procedure is depicted in Table 1.

**Table 1:** Steps in the Knowledge Translator procedure.

**Step #**	**Step description**
1	Read a rule.
2	Extract confidence, category, and other components from the rule.
3	Split the rule's hypothesis into partial cedents.
4	Parse each partial cedent and create an object representing the cedent.
5	Develop an explanation for each partial cedent.
6	Create a rule object containing the cedent objects and explanations.
7	Encode that rule object to a file in KB.

#### 2.2.2. Inference engine

With knowledge encoded in the form of “if-then” rules, an automatic inference component is used to control the application of the rules. Each rule has a left-hand side (“if” statement—the antecedent of a rule) and a right-hand side (“then” statement—consequent of a rule). The left-hand side contains information about certain facts about the patient. If the left-hand side of the rule (antecedent) is matched, its right-hand side (consequent) is executed. Once a new patient's characteristics are entered into the system, the inference module will fire the matching rules from its knowledge base. Consequent clauses decide on the diagnosis/treatment decision suggested to the physician. The JESS library for Java-based programs was used to implement an inference engine based on the efficient pattern-matching algorithm, called Rete (Forgy, 1982).

### 2.3. Graphical user interface

The user interface (UI) of the system constitutes the mode of interaction between the physician and the underlying CDS model. The prototype GUI was developed in Java Swing, with customary component extensions for screen development. The developed UI supports clinical processes in

- Storing and managing the data related to
  – Tinnitus patients—demographics, medical history, audiology evaluations, and structured interviews (Jastreboff and Jastreboff, 1999);
  – TRT visits—diagnoses, treatment applied (sound therapy and counseling), and the outcome evaluation with standardized forms, such as *Tinnitus Handicap Inventory* (THI) (Newman et al., 1995) and *Tinnitus Functional Index* (TFI) (Meikle et al., 2011).
- Providing evidence-based diagnostic and treatment decision support with explanations and quantifiable predictive outcomes

Prior to designing the appearance of the user interface, several factors were taken into account. For this process, several ideas explored in Carroll et al. (2002) were considered. The article proposes the following guidelines for designing a user interface for clinical decision support systems, which provided a basis for research methods for effective GUI design for CDSS for tinnitus:

- “All clinical data should be represented clearly in a format familiar to clinicians and easily understood by patients.”
- “The system should be easy to learn and navigate around.”
- “All information processing should be ‘invisible' to the user.”
- Consider both the physician and the patient as primary stakeholders.
- Use visual aids to describe data such as sliding bars and color codes, where applicable.

## 3. Results

Within this section, results on feature selection, machine learning experiments, and the evaluation of KB and CDSS are described.

### 3.1. Feature selection

The feature selector used in WEKA was used based on a chi-square measure to identify a subset of most predictive attributes. Table 2 shows the results of feature selection, from around 603 attributes that describe the TRT visits dataset (questionnaires, interviews, audiology, and pharmacology). Audiological measurements were indicated as the most relevant factors in the TRT categorization process. The results point out various audiological measurements, such as loudness discomfort level (LDL), the threshold of hearing (Th), and loudness match as primary in relation to classifying (diagnosing) patients into categories.

**Table 2:** Feature selection results for categorizing patients based on chi-squared ranking in WEKA.

**Feature**	**Feature description**	**Ranking score**
LR4	LDL (RE) at 4 kHz	725.1
Th L	Hearing threshold (LE)	712.7
LR3	LDL (RE) at 3 kHz	688.0
LR2	LDL (RE) at 2 kHz	683.4
LR1	LDL (RE) at 1 kHz	683.1
T LR	Tinnitus Loudness Match (RE)	672.6
LR8	LDL (RE) at 8 kHz	670.57
LL3	LDL (LE) at 3 kHz	667.47
Th R	Hearing threshold (RE)	618.94
LL2	LDL (LE) at 2 kHz	617.06

### 3.2. Machine learning models

WEKA was used to test different classification algorithms and determine the classification model with the highest accuracy. The evaluation was carried out by splitting the dataset into training and test subsets using cross-validation with 10 folds. Performance measures for predictive models include classification accuracy (the percentage of correctly classified patients) and precision (how many of the predicted categories are actually in that category). Preliminary results of predictive models with different algorithms are presented in Table 3. The tests were performed on different types of datasets and using different feature selection methods. *Pat-vis* is a dataset with each visit of a patient as a separate instance. *Pat-vis-med* dataset additionally includes binary attributes for all types of medications, that is, each visit instance is repeated for a medication that a patient is taking. *Pat-vis0* includes only initial visits (Visits with ordering number 0 or 1), that is when the diagnosis and categorization of a patient are decided by a clinician. Depending on the feature selection method chosen, the dimensionality of datasets (# features) was reduced accordingly. ML algorithms tested included tree-based (J48), random forests, and Naive Bayes. The most reliable results were obtained using the dataset with the initial visit only, but due to the reduction in the number of data instances, the best accuracy was 57.4% with the Naive Bayes. It is expected that once more data on initial visits is collected, the more precise the trained models become.

**Table 3:** Results on patient classification using WEKA using different data pre-processing, feature selection, and algorithms.

**Dataset**	**# instances**	**# features**	**J48 (%)**	**Naive Bayes (%)**	**Random forest (%)**
Pat-vis-med	6,991	80	88.5	75.2	**89.3**
Pat-vis-med	6,991	20	**87.5**	81.5	87.1
Pat-vis	3,125	603	70.2	55.4	**71**
Pat-vis	3,125	488	69.7		
Pat-vis01	1,090	603	**52.1**	46	49.2
Pat-vis0	599	603	43.2	52	**53.4**
Pat-vis0	599	100	41.0	**57.4**	49.2

The best results are in bold.

### 3.3. Rule mining

The results described in this section include results from rule mining with LISp-Miner 4ft-Miner (association rules) and Act4ft-Miner (action rules) (Simunek, 2014).

#### 3.3.1. Association rules for diagnosis

Experiments on decision rule discovery were carried out to complement results on predictive models for diagnostic decision-making. The variables investigated included 593 variables describing the patient and their tinnitus. Audiology variables include a pure-tone audiogram (up to 12kHz) and the determination of pure tone loudness discomfort levels (LDL) measured for all frequencies in the audiogram. For example, R6 describes the right ear (R) pure-tone threshold for 6kHz. LDL is the audiological measure crucial for TRT diagnosis. For example, LR1/LL1 describes LDL for the right ear/the left ear tests with 1 kHz. Patients' responses to initial/follow-up questionnaires are another important source of information for determining the category in TRT. The questions provide a structure for the interview with a patient and allow physicians to track the progress of the treatment. Variables describing subjective tinnitus are measured on a Likert scale (0–10) and patients are asked to assess them “on average over the last month”.

Table 4 shows examples of extracted associations between audiological measurements, questionnaire responses, and a category of a hearing problem.

**Table 4:** Examples of discovered decision rules for the category of a hearing problem determined based on the interview and audiometric values.

**Sample association rule for diagnostics in TRT**	**Confidence (%)**
*R*3(< 15;20)) ∧ *TAn* ≥8 ⇒ *Category*(1)	94
*H*_*pr*_(< 0;0.5)) ∧ *HL*_*pr*_(< 0;0.5)) ∧ *T*_*pr*_(< 6;8)) ⇒ *Category*(1)	85
*L*_2_ ≥ 50 ∧ *R*_6_ ≤ 75 ⇒ *Category*(2)	87
*Norvasc*(*yes*) ∧ *Tside*(*yes*) ⇒ *Category*(2)	67
*T*_*pr*_(< 0;2.5)) ∧ *H*_*An*_(< 1.5;3.5)) ∧ *H*_*Sv*_(< 1.5;3.5)) ⇒ *Category*(3)	83

These rules are interpreted as follows:

- *If* an audiometric value of *R*_3_ (audiogram at 3 kHz for the right ear) is in the range < 15;20) and annoyance over tinnitus *T*_*An*_ is greater than or equal to 8, *then* a patient falls under Category 1 with 94% confidence.
- *If* hyperacusis *H*_*pr*_ and hearing loss *HL*_*pr*_ are not indicated as problems, but tinnitus *T*_*pr*_ indicated a problem—*then* a patient falls under Category 1 with 85% confidence.
- *If* an audiometric value of *L*_2_ (audiogram at 2kHz for the left ear) is greater or equal to 50 and *R*_6_ (audiogram at 6kHz for the right ear) is less or equal to 75, *then* a patient falls under Category 2 with 87% confidence.
- *If* a patient was taking *Norvasc* and tinnitus was its side effect, then a patient falls under Category 2 with 67% confidence.
- If the score for tinnitus as a problem *T*_*pr*_ was in the range < 0, 2.5), annoyance over hyperacusis *H*_*An*_ in range < 1.5;3.5) and severity of hyperacusis *H*_*Sv*_ in range < 1.5;3.5), then a patient is categorized into Category 3 with 83% confidence.

In general, many such rules are generated and each rule represents a small chunk of knowledge available through a clinical dataset. For example, patients in Category 1 have a significant tinnitus problem (*T*_*Pr*_—Tinnitus as a Problem) but without hyperacusis (*H*) and there is no significant hearing loss (*HL*). Category 2 is characterized by a significant hearing loss, as indicated by lower values of the pure-tone audiogram (*L*_2_ and *R*_6_). Patients in Category 3 are on the other hand characterized by a significant hyperacusis problem (*H*_*An*_—Hyperacusis annoyance and *H*_*Sv*_—Hyperacusis severity). The experiments also yield novel and unknown patterns such as dependencies between certain medications and their side effects (*T side*) being tinnitus symptoms. Experiments between demographics of patients and a TRT category indicated, that tinnitus in elderly patients was frequently related to hearing loss and was affected by many other medical conditions, such as hypertension and age-related afflictions, and associated with Category 2. Patients in Category 1 (C1) were middle-aged, and their tinnitus was associated with psychological disorders, such as depression, anxiety, and panic. Category 3 was frequent in the younger group (30–38 years) and association rules indicate, for men: background in noise exposure, occupation, type of work; and for women: background in stress and hormonal therapy. These findings lead to a hypothesis that a personalized approach to tinnitus treatment based on a patient's profile could be effective. For example, for C1-patients personalized counseling is expected to be more effective, as it is frequently associated with psychological disorders. C2 would be most effectively treated with hearing aids and instrument fitting, as it is frequently associated with hearing loss.

#### 3.3.2. Action rules for recommending treatment

Action rules are methods proposed within this research to support treatment within TRT protocols. The attribute used as a decision attribute is THI's total score (*T sc*), which keeps track of the treatment progress. In case the total score is missing in the data, the tinnitus awareness score (*T*_*aw*_) was used instead. The action rule mining was set up to extract patterns that bring changes in THI's total score/tinnitus awareness for the better (*ChTsc*/*ChTaw*-change in the total score/awareness from the previous visit and *PerChTsc*/*PerChTaw*—percentage change of the previous). The action rule mining experiments involved checking variables related to changeable (“flexible”) treatment methods within TRT and setting other attributes as “stable” (patient demographics, tinnitus characteristics), with the goal to improve metrics measuring the severity of tinnitus. Sound therapy with instruments involves choosing the right instrument and fitting the instrument with the optimal setting over time at subsequent visits. There are different types of instruments, as described by the category variable (*Ins*): hearing aid (*HA*), sound generator (*SG*), and combination instrument. There are different SG models, e.g., General Hearing Instrument (*GHI*): soft/hard, Viennatone (*V*), and many others. A specific fitting of instruments is a significant aspect and real-ear measurements (REM) assist in instrument fitting. Sound therapy is accompanied by counseling. The variable FU describes the types of follow-up contact: audiology and counseling (A), counseling (*C*), telephone-based (*T*), and e-mail based (*E*). The results of the sample extracted patterns are presented in Table 5.

**Table 5:** Results on actionable knowledge discovery for recommending treatment in TRT.

**A sample treatment action rule**	**Conf. (%)**
*G*(*m*) ∧ *NTI*(*yes*):(*Ins*_*vis*(01)_(*GHH*) → *Inst*_*vi*(01)_(*GHS*)) = > *Ch*(*better*)	80
*T*_*side*_(*yes*) ∧ *OMTI*(*yes*):(*Ins*_*vis*(01)_*GHH* → *V*) ∧ *FU*(0 → *T*) ⇒ *Ch*(*better*)	82
*Ins*(*SG*):(*Mix*_*RSL*_(< 11;12) → < 9;10)) ⇒ *Ch*(*better*)	100
*FU*(*A*) ∧ *Ins*_*vis*(01)_(*GHI*) ∧ *Freq*_*LE*_(< 3000;3150)):(*treat*(< 5;6) → < 6;8)) ⇒ *Ch*(*better*)	88

The rules present different actions in treatment leading to a change in patients for the better, as measured by the total score of THI and tinnitus awareness. These rules are interpreted as follows:

- *If* a patient is a male and tinnitus is noise-induced *then* changing sound therapy from the instrument model of GH hard (*GHH*) to GH soft (*GHS*) at the first visit improves a patient with 80% confidence.
- *If* tinnitus was induced by another medical condition (*OMTI*) and as a side effect of taking medications (*T*_*side*_), *then* changing the sound generator model GH hard (*GHH*) to the Viennatone model (*V*) at the first visit and changing the follow-up contact to the telephone-based (*T*) improves patient with 82% confidence.
- *If* the current treatment involves sound generator *SG, then* changing the mixing point for the right ear *Mix*_*RSL*_ from < 11;12) to < 9;10) improves a patient's state with 100% confidence.
- *If* the current treatment involves audiology (*FU*(*A*)) with the GHI instrument and frequency in the left ear measured by REM -*Freq*_*LE*_—in the range of < 3000;3150) *then* prolonging that treatment from 5–6 weeks to 6–8 weeks brings improvement with 88% confidence.

The extracted rules offer high precision, e.g., how to fit a particular model of a particular type of instrument (*Ins*(*SG*):(*Mix*_*RSL*_(< 11;12) → < 9;10))) or how to change the length of treatment with a specific method [e.g., *treat*(< 5;6) → < 6;8]: change the length of treatment from 5–6 to 6–8 weeks. This approach also offers high personalization: the treatment actions leading to improvement are extracted for the individual patients' profiles, as described by demographics [e.g., *G(m)*- gender: male] and the tinnitus background (e.g., *NTI*- noise-induced tinnitus, *OMTI*- other medical-induced tinnitus, *T*_*side*_ - tinnitus as a side effect of pharmacology).

### 3.4. Knowledge translator

The Knowledge Translator was tested within CDSS for efficiency and scalability. The runtimes of various steps of the Knowledge Translator are depicted in Table 6. The encoding step encompasses all operations from reading the files with rules to writing the rules into KB. Parsing, in this case, only refers to parsing the rule and creating an object in memory, but it does not include any I/O operations. The tests were run using 2,192 diagnosis rules and 1,348 treatment rules. An average from running one test 5 times is presented in Table 6.

**Table 6:** Runtimes for encoding and parsing diagnosis (total of 2,192 rules) and action rules (total of 1,348).

**Rule type**	**Total encoding time (s)**	**Total parsing time (s)**	**Time to parse a rule (ms)**
Diagnosis rule	0.29	0.22	0.098
Treatment rule	0.24	0.13	0.094

**Figure 5:**
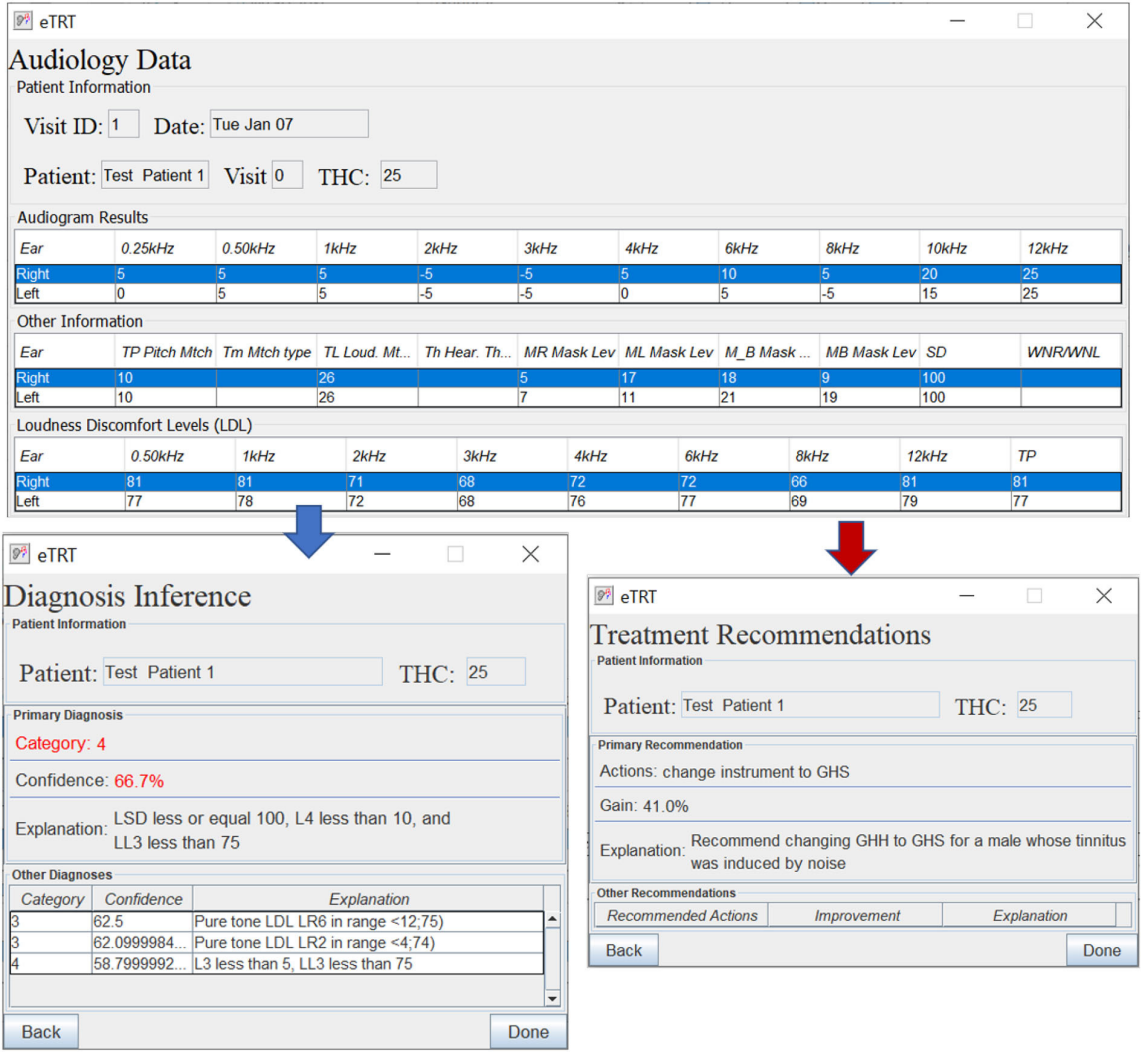
The diagnostic/treatment inference results for test case 1 (noise-based, middle-aged male) based on audiometry: (1) primary diagnosis of category 4 with 66.7%, and (2) treatment recommendation for changing the instrument type with the expected decrease in tinnitus severity by 41% points.

As one can see from the results in Table 6, the developed Knowledge Translator encodes and parses a massive amount of extracted rules in a relatively very short time. This provides an important step in the future scalability of the CDS system. When comparing the time to parse a single rule by the Knowledge Translator (less than 0.1 ms) vs. the same task performed manually (manual encoding by a human, which takes approximately 2 min at least to read, interpret and encode a rule in a correct syntax), the time gain is enormous. Additionally, the developed Knowledge Translator encodes the human-understandable explanations, which are critical for clinical use and support in the accurate diagnosis of the category of a hearing problem and treatment actions recommendations (refer to Figure 5).

### 3.5. CDSS evaluation

**Figure 6:**
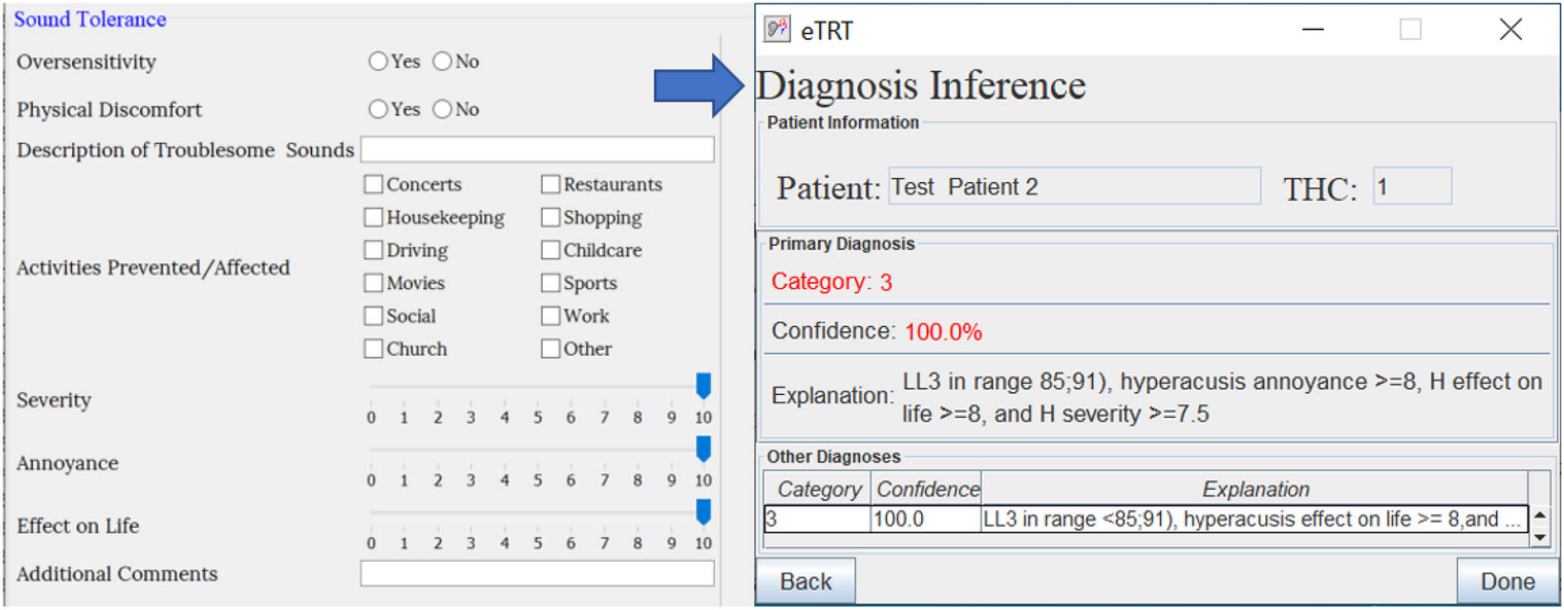
The diagnostic inference for test case 2 based on audiometry/initial interview. The explanation for Category 3 with 100% confidence included a high score for hyperacusis as a problem.

**Figure 7:**
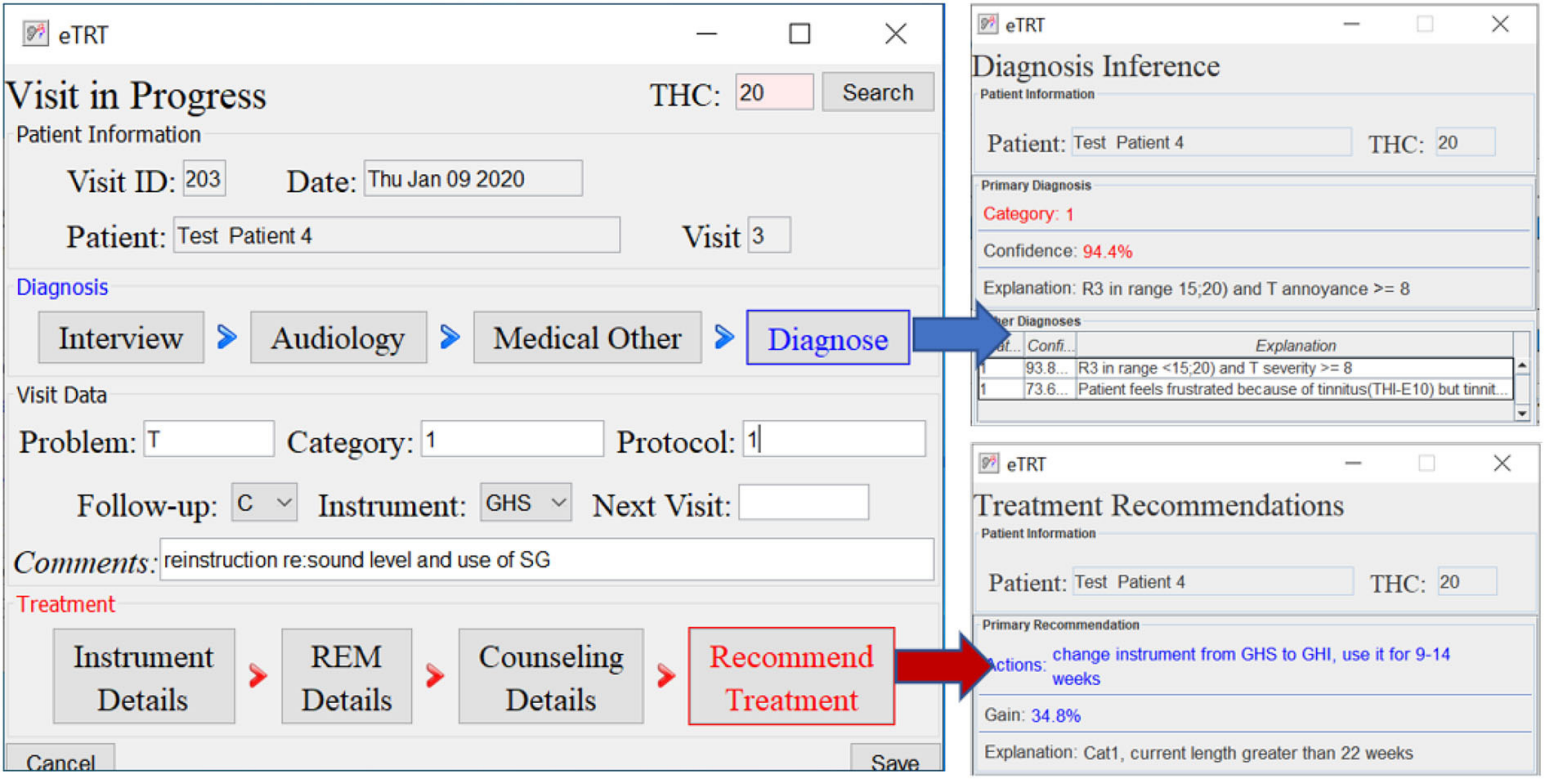
The diagnostic/treatment inference results for test case 4: (1) category 1 was inferred based on the audiometry results and initial interview (annoyance over tinnitus high); (2) recommendation included the change of the sound instrument from GH soft and shorten its application time to 9–14 weeks with an expected gain of 34.4% points.

The evaluation study was to determine whether the built CDS system does what it was intended to and at an adequate level of accuracy. The expectation from the proposed CDSS is to generate accurate, patient-specific, and interpretable clinical suggestions. This will encourage efficient and effective use of tinnitus retraining therapy for the management of hearing disorders. The evaluation study involved:

1. Developing a user-friendly interface to input the patient's data.
2. Identifying a set of representative test cases of patients from the dataset not used for building the model.
3. Running inference on the chosen test cases entered into the system (refer to Figures 5–7).
4. Performing quantitative and qualitative evaluation of the system based on the results from the above.

The metrics used for this evaluation of the system include:

- Accuracy—The number of correct predictions vs. the total number of predictions. To compute the accuracy we compare the system's recommendations with the actual diagnosis/treatment decision made by a physician.
- Coverage—The number of test cases matched against the knowledge base.
- Interpretability—If the recommendations are explainable and understandable by humans.

#### 3.5.1. Test cases

The representative cases from each of the 5 categories, were identified. Future testing will involve identifying more cases per category. The chosen test cases reflect the heterogeneity of the hearing problem and patient profile; a test patient for each etiology and each category of the hearing problem was identified from the tinnitus patient database (refer to Table 7).

**Table 7:** Patient test cases—patient profile, etiology of their hearing problem, the diagnosed category, and the treatment protocol determined by the physician.

**Test case**	**Patient profile**	**Etiology**	**Diagnosis**	**Treatment protocol**
1	Male, age 38, KY	Noise exposure	Category 4	Category 4
2	Male, age 49, GA	Ear surgery	Category 3	Category 3
3	Female, age 77, FL	Hearing loss	Category 2	Category 2
4	Male, age 53, GA	Stress-related	Category 1	Category 1
5	Male, age 36, GA	Car accident	Category 0	Category 1

Tables 8, 9 provide the diagnostic and treatment inference results for all test cases.

**Table 8:** Results on predicting diagnosis by the system on the chosen patient test cases—actual category vs. category predicted by the system, characterized by confidence, and explanation.

**Test case**	**Actual**	**Prediction**	**Conf**.	**Explanation**
1	Cat 4	Cat 4	66.7%	LSD < = 100, L4 < 10, and LL3 < 75
2	Cat 3	Cat 3	100	LL3 in < 85;91), Hyper. Annoy ≥ 8,
				H Eff on Lif ≥ 8, and H Sev ≥ 7.5
3	Cat 2	Cat 2	96.2	LR8 ≥ 999, R6 ≥ 75, and *T*_*sv*_≥ 8
4	Cat 1	Cat 1	94.4	LL3 in < 15;20) and Tin. annoy. ≥ 8
5	Cat 0	Cat 1	60.3	A patient often irritable by tinnitus (E14)
				and tinnitus makes him anxious (E22)

**Table 9:** Results on recommending treatment actions, characterized by an expected improvement gain in percentage points and explanation(s) for the patients' test cases.

**Test case**	**Recommended action(s)**	**Gain**	**Explanation**
1	Change instrument from GHH to GHS	41 pp	A male whose tinnitus was induced by noise
4	Change instrument from GHS to GHI,	34.8 pp	Cat1, instr. duration
	use it for 9–14 weeks		greater than 22 weeks
5	Change Freq LE from < 2,800; 3,000) to < 2,670; 2,800) in REM	8.4 pp	Instrument used GHS

#### 3.5.2. Diagnostic decision support

The diagnosis prediction was 80% accurate and covered 100% of cases (refer to Table 8). The average confidence in the primary diagnosis inference was 83.51%. The only incorrect prediction was for test case 5. After closer investigation, this case was annotated by the physician as a “discrepancy in information” in interview data, and “inconsistent results” in audiological evaluation, which are the reasons that misled the predictive model (as an “outlier” data point). Moreover, the actual protocol followed was the same as for the category predicted by the system.

#### 3.5.3. Treatment decision support

The treatment recommendations were generated for 3 out of 5 patient test cases (refer to Table 9). The other two cases were not covered, that is, no action rule was matched with the patient profile, due to a limited number of rules encoded manually in KB at the time of testing.

For all the tested cases, both the diagnostic and the treatment recommendations were explained with a human-comprehensible message/reason. The explanations were provided by means of the premises of the rules in KB that were matched against the current patient's profile/visit data. The predictions' probabilities were quantified by means of the matched rules' confidence metric.

## 4. Discussion

Data mining is an active field and helps uncover links between variables, with the goal to develop optimal strategies for tinnitus management. This will open new horizons for TRT, which does not have a stagnant protocol but continues to evolve based on information gathered from treatments of patients (Jastreboff, 2015). TRT has successfully been used in a clinical setting to help patients with tinnitus and decreased sound tolerance since 1988, but the method of TRT underwent many modifications since its first description.

### 4.1. Strengths

The proposed analytical models applied to a clinical dataset detected both trivial and known patterns in TRT, but also unexpected, unknown, and potentially interesting patterns. The extracted knowledge utilizes clinical knowledge available from the TRT expert through the dataset. The prime characteristic of the approach is its capability of expressing knowledge in a linguistic way allowing a system to be described by simple “human-friendly” rules. Knowledge represented in form of rules is closely related to human thinking and can be explained natively. It also offers an approach for modeling the uncertainty and the imprecision typical of human reasoning. As knowledge is created based on feedback from an expert, users can also rely on it. It can be used for educational purposes as a training tool to spread expertise in TRT. The knowledge once encoded will be preserved permanently and utilized on any hardware. New patient data can be analyzed by inferring from rules with the goal of providing a prediction of optimal diagnosis and treatment. This computer-based approach will help clinicians reveal the mysteries of tinnitus heterogeneity and decrease the impact of this major health problem on the patient and society.

### 4.2. Limitations

The currently identified limitations include limited access to clinical TRT datasets and a lack of standardization in data management techniques among clinics offering TRT. The more data, including from many providers, and the more recent the data (since TRT is still evolving) the more accurate and useful the CDSS tool. The key to the successful adoption of the system is a collaboration between the system's developers and clinicians/clinics. At the current stage, mostly the prototype version was designed and developed, which nevertheless has to be tested for usability in real-world environments and undergo rigorous testing.

The main so-far identified problem is the quality of the data, and particularly its sparsity. Data is available from only one expert, which provides consistency of knowledge, but also limited records of data. Generally, the more data, the more accurate the results. Missing values and a limited amount of data results in extracting rules characterized by relatively low support and confidence. Additional strategies will be investigated to develop algorithms for the reliable imputation of missing values. Another potential problem is the computational complexity of learning analytical models. The strategy to handle this issue is to investigate alternative efficient algorithms for mining that make use of multiple cores and distributed processing. Additionally, the hardware platform will be scaled adding RAM and CPU power.

The validation of CDSS accuracy is a challenge. System reliability and trustworthiness depend on the quality of the rules. In the current testing design, retrospective patient data will be used to design and test the system. CDS logic may not precisely fit the patient. Therefore, if coverage results prove to be unsatisfactory, more rules will be extracted/added. Another potential problem is that the user interface proves to be unsatisfactory. In that case additional, alternative UI designs will be proposed, evaluated, and compared. If the attractiveness of the interface will be insufficient, alternative technologies for UI development will be investigated, e.g., Java FX.

### 4.3. Future study

In the future, the system is to be used as a TRT assistant by medical professionals to support both the efficient and effective management of tinnitus.

The tasks to be performed in the longer term, related to the development of the CDS system include:

- Expanding the knowledge base with new clinics and new/updated treatment methods. Adding new data sources to the system, such as patient and treatment datasets from other clinics, to expand the knowledge base of the system. Both clinics in TRT as well as other tinnitus treatment methods should be included. The goal central repository should be made available to participating TRT clinicians and researchers (Landgrebe et al., 2010). Additional approaches for tinnitus treatment will be investigated, such as music therapy, brain stimulation, or cognitive therapy. It is expected more data available from more than one TRT expert will improve the accuracy and coverage of the knowledge base in TRT.
- Applying machine learning methods to investigate additional factors and variables that may help understand and treat tinnitus. Some of the considered factors include magnetic resonance imaging (MRI) data, e.g., to understand how sound therapy changes neuronal activity and modulates the brain network (Han et al., 2019); or associations between gene variants and tinnitus states (Pulley et al., 2012).
- Expanding AI-based methods to provide decision support, i.e., natural language processing from the clinical text data, i.e., doctor's comments (Tarnowska and Ras., 2019, 2021). Another potential is to utilize natural language understanding to develop conversational agents, that can help in delivering counseling to tinnitus patients. Additionally, machine learning methods based on clustering techniques to develop new models for more personalized treatment can be investigated.
- Integration with other software used in audiology (Rajkumar et al., 2017), i.e., software for sound generators' tuning; investigating computer methods to generate personalized sound used in tinnitus habituation (Barozzi et al., 2017); integrating music therapy and music recommendation into the system (Tarnowska, 2021).
- Expanding modes for the system—i.e., publicly-available touch-screen stations or developing mobile applications to improve personalization and streamline data collection from the patients (Blome, 2015).

The long-term goal of this research is to deploy such a system in a clinical setting to enhance health-related decisions in TRT delivery. This step will be preceded by testing the system in the clinical environment and testing its usability within real physician-patient consultation. More extensive testing involving more test cases and new patients will be conducted in the future. Usability evaluation with actual clinical users should be performed to determine its acceptability. In the future, the system should be integrated with health IT systems and electronic health records (EHR) to fit into the workflow of clinical decision-making. The electronic health record (EHR) with embedded clinical decision support is recognized as an important component in providing improvement in patient safety, healthcare quality, and efficiency, as promised by HITECH (Health Information Technology for Economic and Clinical Health) policy initiatives (Blumenthal and Glaser, 2007). The project is intended to connect primary care providers and TRT specialists using a knowledge-driven computational engine that aids in diagnosing and planning treatment for tinnitus patients. Decision support, delivered using an information system with the electronic medical record as the platform, will provide decision-makers with tools making it possible to achieve large gains in performance, narrow gaps between knowledge and practice, and improve tinnitus habituation rates. The proposed novel and efficient approach to developing a data-driven CDSS can be applied to various other medical domains. The results are replicable by others, and useful to tinnitus researchers and other medical practitioners.

## 5. Conclusion

The main contribution of this study is proposing and evaluating a data-driven clinical decision support system to assist audiologists in the diagnosis and treatment of hearing disorders, such as tinnitus, hyperacusis, and misophonia. Up to date, no CDSS specialized in tinnitus diagnosis and therapy has been designed and implemented. Collaboration between experts in the fields of both data analysis and tinnitus is of utmost importance to prepare and validate optimal CDSS that will be reliable and efficient. Such decision support will bring advantages such as speed, accuracy, and long-term storage of information. Medical users will receive rapid and synchronous advice. With the user-friendly interface, non-computer professionals will be able to easily operate the system and interpret its results. Documented knowledge can be used for future training and educational purposes. This type of research is expected to provide an important step toward the widespread and effective use of TRT knowledge in clinical practice. This is significant because the diagnosis and treatment of TRT is a complex task. It requires a very high level of expertise to operate accurately and efficiently. Data and information being used in tinnitus management are becoming heterogeneous and large in volume, and therefore, they are overwhelming. A CDSS needs to be developed once and customized locally to the clinic's needs. It can be used frequently in many places by many people without location restrictions. The system offers a scalable architecture that can be extended by new knowledge.

## Data availability statement

The raw data supporting the conclusions of this article will be made available by the authors, without undue reservation.

## Author contributions

ZR and KT: study conception and design. PJ: acquisition of data. KT, ZR, and PJ: analysis and interpretation of data. KT: drafting of the manuscript. ZR and PJ: critical revision. All authors contributed to the article and approved the submitted version.

## Conflict of interest

The authors declare that the research was conducted in the absence of any commercial or financial relationships that could be construed as a potential conflict of interest.

## Publisher's note

All claims expressed in this article are solely those of the authors and do not necessarily represent those of their affiliated organizations, or those of the publisher, the editors and the reviewers. Any product that may be evaluated in this article, or claim that may be made by its manufacturer, is not guaranteed or endorsed by the publisher. All claims expressed in this article are solely those of the authors and do not necessarily represent those of their affiliated organizations, or those of the publisher, the editors and the reviewers. Any product that may be evaluated in this article, or claim that may be made by its manufacturer, is not guaranteed or endorsed by the publisher.
